# Translation and cross-cultural adaptation of the Model Disability Survey (MDS) for Brazil

**DOI:** 10.11606/s1518-8787.2023057004759

**Published:** 2023-06-02

**Authors:** Érika Giovana Carvalho da Silva, Shamyr Sulyvan Castro, Carla Sabariego, Karolinne Souza Monteiro, Núbia Maria Freire Vieira Lima

**Affiliations:** I Universidade Federal do Rio Grande do Norte Faculdade de Ciências da Saúde do Trairi Santa Cruz RN Brasil Universidade Federal do Rio Grande do Norte. Faculdade de Ciências da Saúde do Trairi. Santa Cruz, RN, Brasil; II Universidade Federal do Ceará Departamento de Fisioterapia Fortaleza CE Brasil Universidade Federal do Ceará. Departamento de Fisioterapia. Fortaleza, CE, Brasil; III University of Lucerne Department of Health Sciences and Medicine Lucerne Switzerland University of Lucerne. Department of Health Sciences and Medicine. Lucerne, Switzerland

**Keywords:** Disability Evaluation, International Classification of Functioning, Disability and Health, Surveys and Questionnaires, Translations, Cross-Cultural Comparison, Reproducibility of Results

## Abstract

**OBJECTIVE:**

This study has as objective the translation and cross-cultural adaptation of the Model Disability Survey (MDS), a World Health Organization instrument that provides comprehensive information on disability/functioning, for Brazil.

**METHODS:**

This is a cross-sectional methodological study, carried out through five stages – initial translation, synthesis of translations, reverse translation, review by a specialist committee, and pre-test –, considering properties such as semantic, idiomatic, experimental, and conceptual equivalence. Translators, researchers, a mediating team, health professionals, a methodologist and a language specialist were needed to pass through the stages. Statistical analysis was produced from absolute and relative frequencies, measures of central tendency and dispersion, normality tests and content validity index (CVI) > 0.80.

**RESULTS:**

The MDS has 474 items, which generated 1,896 analyzes of equivalence. Of these, 160 items had a CVI < 0.80 in at least one of the four types of equivalence and required adjustments. After adaptations and approval by the judges, the pre-final version went on to the pre-test with 30 participants from four regions of the Brazilian Northeast. Regarding this sample, 83.3% are women, single, with an average age of 33.7 years (SD 18.8), self-declared as black or brown, active workers, with technical education and living with three residents. Interviews lasted 123 minutes on average, where 127 health conditions were mentioned, and the most frequent cited were anxiety and back pain. Answers were analyzed and 63 items were cited as needing some adjustment, two of which were submitted for analysis by the committee because they presented a CVI < 0.80. The instrument, guide and presentation cards were adjusted after a new pre-test.

**CONCLUSIONS:**

The MDS was translated and cross-culturally adapted to Brazilian Portuguese and showed adequate content validity.

## INTRODUCTION

In 2011, the World Health Organization (WHO) estimated that more than one billion people, equivalent to 15% of the world’s population, have a disability^1^ . In Brazil, according to data from the 2019 National Health Survey (PNS), 17.2 million people aged 2 years or over (8.4% of the population) have some type of disability, and of these 8.5 million (24.8%) are older people^2^ .

The existing worldwide data on disability need to be standardized, given that, to date, no gold standard instrument has been used for collecting data that provides comprehensive and systematic documentation on the subject^1 , 3^ . In addition, it is understood that the limited number of questions included in censuses, such as that in Brazil, is not sufficient to measure the number of Persons With Disabilities (PWD), and that, in this scenario, the use of cross-culturally adapted and validated standardized instruments is essential to fill this gap^4^ .

In this sense, it can be assumed that it is necessary to develop standardized methodologies for collecting PWD data, in line with cultural aspects and consistently applied, which allow international comparisons and monitoring of progress with regard to public policies^5^ . The World Report on Disability emphasizes the importance of countries in general being aware of the number of existing PWD, as well as their life contexts, with a view to adapting the provision of services and making them more efficient^1^ .

In Brazil and in the world, there are measurement instruments aligned with the biopsychosocial model, translated and cross-culturally adapted, which aim to measure functionality, including the International Classification of Functioning, Disability and Health (ICF)^6^ checklist, the Core Sets, the World Health Organization Disability Assessment Schedule (WHODAS 2.0)^7^ , and the Brazilian Functioning Index (IFBr).

The ICF checklist is a generic instrument for health conditions that measures functionality, but the limitation of codes that can be used by each interviewee is a negative point for its application^6^ . The Core Sets are summarized lists of ICF codes with application to specific health conditions, in a quick and easy manner; however, they focus the assessment on the disease/health condition. The WHODAS 2.0 is a generic instrument that assesses the perceived disability associated with the health condition, and it is quick to apply. Nevertheless, it was not created for population surveys and does not have cut-off points for levels of disability^7^ .

The IFBr is an instrument proposed by the Brazilian government in 2011 with the objective of identifying external factors that can influence the individual’s life and how much they can impact on their functionality. It generates a score that classifies the individual’s level of dependence or functional independence as mild, moderate, and severe^8^ . Faced with the need for some changes in the IFBr, the Adapted Brazilian Functioning Index (IFBrA) was created, which is used to assess the need for retirement, and its use is restricted to adults with disabilities active in the labor market^11^ .

Another point to be highlighted is the importance of using a standardized tool for collecting population data on the impact of disability on people’s lives, thus avoiding data collection with discordant or mistaken understandings regarding functionality^6^ . Especially for countries with equity-based health systems, knowing how many people have disabilities is not enough to determine their health needs; for this, data on disability are needed^12^ .

Given the above, the Model Disability Survey (MDS) emerged from a World Bank-WHO partnership^13^ . This instrument has two versions, comprehensive and summarized, both standardized for data collection in surveys at the population level, which provide information about how people spend their lives and the barriers they encounter, taking into account environmental and personal factors, capacity and performance, allowing comparisons between groups with different levels of functionality^14 , 15^ .

The MDS has already been implemented through national surveys in Chile (2015), Sri Lanka (2015), Philippines (2017), Qatar (2017), Costa Rica (2018) and Afghanistan (2019), regionally in Cameroon (2016), Pakistan (2017) and United Arab Emirates (2018), and also through pilot studies in Cambodia (2014), Malawi (2014) and Oman (2016), thus it has been translated into Arabic, Spanish, Sinhalese, Filipino, French, Khmer, and Dari. However, one has found no studies detailing the MDS translation and cross-cultural adaptation procedures in these countries^13^ . In a study with populations from Chile and Sri Lanka, the instrument (short version) revealed valid metrics to measure disability^16^ .

The MDS is based on the theoretical basis of the ICF^17^ , whose construction facilitates health surveys that compare data on disability at the international level^18^ . The results from national surveys allow and guide the planning and development of public policies aimed at the full social integration of PWD^19^ . In this context, the objective of this research was to make the translation and cross-cultural adaptation of the comprehensive version of the MDS for Brazil, as well as analyzing the content validity of this Brazilian version.

## METHODS

This is a study on MDS cross-cultural translation and adaptation to Brazilian Portuguese, developed by the Research and Innovation Network in Sustainable Development Functionality, Health and Goals ( *Rede Fusão* ).

The purpose of the MDS is to collect data on all disability dimensions in order to obtain comprehensive and relevant information that helps countries build a disability portrait, with particular relevance to disability policy; direct and reliable international comparisons of data on the subject; and national and global monitoring of the implementation of the International Convention on the Rights of Persons with Disabilities. The MDS is subdivided into: household questionnaire (cover page, contact data, list of household residents), individual questionnaire (contact data, eligibility, sociodemographic characteristics, professional history and benefits, environmental factors, functionality, health status, personal assistance, assistive devices and facilitators, use of health services, well-being, empowerment, interviewer’s observations), and representative questionnaire (with the same items listed in the previous module)^20^ . The MDS is predominantly used in populations aged 18 and over, although it also has a children module.

The process of MDS translation and cross-cultural adaptation followed the guidelines by Fortes and Araújo^21^ and complied with the Consensus-based Standards for the Selection of Health Measurement Instruments (Cosmin)^22^ recommendations. Besides, it followed the guidelines of Beaton and collaborators^23^ , which provide broad support for the semantic, idiomatic, experimental, and conceptual requirements, establishing five stages, namely: I. Initial translation; II. Synthesis of translations; III. Reverse translation; IV. Review by specialist committee, and V. Pre-test.

The first MDS translation (T1 – stage I) was made by a company specialized in translations, and then the document was revised by the Pan American Health Organization (PAHO) team, representing the translation made with clinical expertise. The second translation (T2) was made by two lay foreign translators with a broad command of Brazilian Portuguese, both not informed of the concepts quantified by the research and without training in the health area.

The synthesis of T1 and T2 (T12 version - stage II) was made by a team of 10 researchers from the health area of the Universidade Federal do Rio Grande do Norte (UFRN), four research professors, one master’s student, and five scientific initiation students. T12 was made via virtual meetings, analyzes and comparative discussions about the questionnaires. In order to ensure the quality of the process, all items were peer-reviewed.

In the reverse translation stage (stage III), T12 was translated into English again (BT1). This translation was made by a bilingual Canadian translator, who has command of Brazilian Portuguese, without knowledge of the original instrument and concepts explored in the research.

Stage IV was based on the criteria of Jasper^24^ to select specialists and considered the minimum composition to be: methodologists, health professionals, language professionals, and translators involved in the process. In this sense, the selection criteria were: being a professional with knowledge of functionality, disability or of the process of translation and validation of health measurement instruments. The specialists were selected from different Brazilian states by consulting their Lattes Curricula of the National Council for Scientific and Technological Development (CNPq), forming a committee with four judges (one of them was a methodologist, and all of them were health professionals) and a specialist in languages. The judges received seven documents with T1, T2, T12, BT1, the MDS in English, the MDS guide in English and Portuguese, schedule of meetings, and other guidelines related to equivalence. These professionals read and analyzed the documents in full with the help of the mediating committee (two professors and a master’s student from UFRN, who participated in the previous stages), and the residual divergences were sent to the language specialist. This mediation team had the main role of guiding the judge committee during the stage, planning and executing strategic virtual meetings, as well as making all necessary adjustments and adaptations to the documents in accordance with the committee’s guidelines.

Each questionnaire item was evaluated using a tool created in Google Sheets, which enabled analyzes being conducted, shared individually with each judge. This tool included all items from the original instrument and from the T12 version, subdivided according to the MDS modules, with qualitative and quantitative fields, one for considerations about translations, adequacy of items and possible suggestions, respectively; and another to classify the semantic, idiomatic, experimental, and conceptual equivalence as adequate translation (AT), partially adequate translation (PAT), and inadequate translation (IT).

In the process of individual evaluation of the items, a Content Validity Index (CVI) greater than 80%^25^ was considered, which refers to the degree to which the content of an instrument reflects adequately the construct measured. The CVI is based on the specialists’ evaluation for each item according to the relevance of the content of an instrument, usually judged by means of Likert scales^26^ . As four members (25% per member)^25^ composed the committee, when one of the judges disagreed with an item, it was reviewed.

The judges’ suggestions were grouped together with their respective justifications and adjusted according to the judges’ consensus. Finally, an integrative seminar was held with the members of this stage through a virtual meeting so that to analyze the instrument in its pre-final version and make adjustments before the pre-test.

For the pre-test stage (stage V), the inclusion criteria for participants were: people over 18 years of age and with the cognitive ability to answer the questionnaire. The exclusion criteria adopted were: refusing to answer all the questions in the questionnaire and withdrawing from the interview before it was completed.

The sample consisted of 30 participants, selected for convenience according to the eligibility criteria and interviewers’ location. Participants were invited by telephone and the interview was conducted in person, in a reserved room. The collection was carried out by eight members of the research project team, composed of professors and students from the UFRN master’s degree courses, who received prior training to carry out the interviews and had knowledge of the study subject.

Answers to the questionnaire were sent by the interviewers to an online database, using Google Forms previously prepared for this stage, in addition to a specific form to assess the interviewee’s understanding. This form was used to record the items that, due to the participant’s report or perception of difficulty observed during the interview, required adjustments to improve understanding. All interviewers were instructed to apply the questions of the comprehension form at the end of each MDS module.

After completing the collection of stage V, all items mentioned in the understanding form were analyzed. For this analysis, the CVI was also considered: % agreement=number of participants who agreed/total number of participants*100. That is, 80%=X/30*100.

In this sense, when there were seven or more citations of disagreement regarding an item in the understanding form (IVC < 80), it was re-analyzed by the specialist committee. The adjusted items were evaluated by the 30 participants through a new pre-test stage, performed only with these pending items, according to a Likert scale of level of understanding, with the following answer alternatives: 1. Very good; 2. Good; 3. Regular quality; 4. Poor; 5. Very poor. At this stage, if there were seven or more citations per item listed as “Poor” or “Very poor,” it was re-analyzed. As the pre-test was done only with the missing items, the interviewers could take this stage via telephone, in order to facilitate the collection process.

The synthesis of all stages of MDS translation and cross-cultural adaptation is presented in the Figure below.

**Figure:**
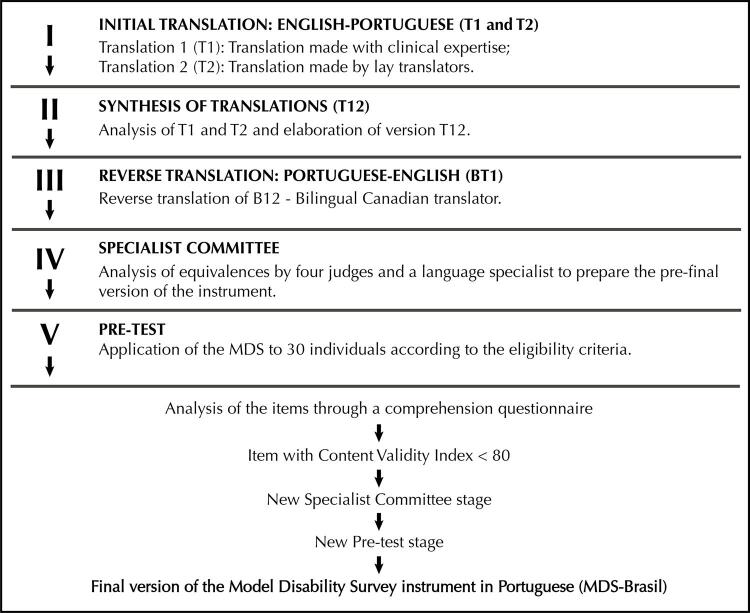
Flowchart of the process of Model Disability Survey (MDS-Brazil) translation into and cross-cultural adaptation to Brazilian Portuguese

The MDS^20^ guide and the presentation cards file were translated as well, which was done by Brazilian professionals with extensive experience in translations and Portuguese teachers, and subsequently submitted to the specialist committee. Therefore, they went through a process similar to that of the comprehensive instrument.

All data collected were entered into a database, created using Microsoft Excel^®^, version 2016, and analyzed using the Statistical Package for Social Sciences (SPSS for Windows)^®^, version 25.0. The sample distribution was presented by means of absolute and relative frequency, and the descriptive analysis was performed using measures of central tendency and dispersion, that is, median and quartiles, respectively. Shapiro-Wilk normality test was applied to verify the distribution normality of the quantitative variables. CVI was used during stages IV and V to measure the proportion or percentage of agreement on the instrument items and establish that the stages were carried out until all items, or a set of items, reached agreement > 0.8026.

All participants in stage V were informed about the research and authorized their participation by signing the Informed Consent Form (ICF). The study complied with the ethical precepts that govern research with human beings in accordance with Resolution No. 466/12 of the National Health Council (CNS), and specified in the Declaration of Helsinki, with the approval of the Institutional Research Ethics Committee of the Faculdade de Ciências da Saúde do Trairi (Facisa) under No. 4.102.958 and Ethical Assessment Presentation Certificate (Caae) No. 31112020.4.0000.5568.

## RESULTS

During the specialist committee stage, the MDS was considered with a total of 474 items, in which statements and module titles count was also included. However, as each item was analyzed according to four types of equivalence (semantic, idiomatic, experimental and conceptual), there were a total of 1,896 analyzes per judge. Table 1 refers to these analyzes of individual agreements carried out by the judges during stage IV.

**Table 1:** Quantitative analysis of concordance of the items verified by the specialists.

Specialist	AT		PAT		IT	

	n	%	n	%	n	%
Judge 1	1,857	97.94%	37	1.95%	2	0.11%
Judge 2	1,773	93.51%	99	5.22%	24	1.27%
Judge 3	1,827	96.36%	66	3.48%	3	0.16%
Judge 4	1,800	94.94%	81	4.27%	15	0.79%

AT: adequate translation; PAT: partially adequate translation; IT: inadequate translation.

A total of 327 equivalents (17.25%) were considered partially adequate and inadequate by the specialists. Taking into account only the divergent items, that is, those in which at least one of the four types of equivalence was classified as PAT or IT by at least one judge, a total of 160 items were sent for discussion with the other judge committee members to deliberation and consensus about the pre-final version.

Due to the large number of items present in the MDS, the Chart illustrates the description of some of those that had some alteration during the final stage of step IV (n = 160), in comparison with the original version and the synthesis of the translations (T12). The changes made are underlined for better visualization.

**Chart:** Comparison of changes in items between the original version and the adapted version of the Model Disability Survey (MDS) for use in Brazil.

Item	Original version	Translation summary (T12)	Pre-final version approved by the committee of judges for application in the pre-test
Module 0200, alternative D	Contact with	Em contato com:	Fez contato com:
Module 1000 of the Household Questionnaire	Household roster	Descrição do domicílio	Lista de moradores do domicílio
Module 2000, Question 2013 of the Individual Questionnaire	Do/did you usually work throughout the year, or do/did you work seasonally, or only once in a while for your main job? Work throughout the year Seasonally or part of the year Once in a while	Em relação ao seu trabalho principal, você costuma/costumava trabalhar durante todo o ano ou apenas em determinadas temporadas ou durante pequenos períodos? Durante todo o ano Determinadas temporadas Ocasionalmente (durante pequenos períodos)	Em relação ao seu trabalho principal, você costuma/costumava trabalhar durante todo o ano, por determinadas temporadas ou durante pequenos períodos? Durante todo o ano Por temporada Ocasionalmente (durante pequenos períodos)
Module 4000, Question 4010 of the Individual Questionnaire	How much of a problem is being clean and dressed?	O quanto manter sua higiene pessoal e vestir-se é um problema para você?	O quanto fazer sua higiene pessoal e vestir-se é um problema para você?
Second statement of Module 4000 of the Individual Questionnaire	Please take into account your health and people who help you, any assistive devices you use or any medication you take.	Por favor, considere a sua saúde e as pessoas que te ajudam, quaisquer dispositivos de auxílio que utiliza ou quaisquer medicamentos que você toma.	Por favor, considere a sua saúde e as pessoas que te ajudam, quaisquer dispositivos assistivos que utiliza ou quaisquer medicamentos que você toma.
Module 3000B of the Individual Questionnaire	Mobility and Self-care	Mobilidade e Cuidado Pessoal	Mobilidade e Autocuidado
Module l6010 of the Individual Questionnaire	Over the last 12 months, did you receive any health care NOT including an overnight stay in hospital, rehabilitation facility or long-term care facility?	Nos últimos 12 meses, você recebeu algum cuidado médico que NÃO necessitaram passar a noite no hospital ou instituição de saúde?	Nos últimos 12 meses, você recebeu algum cuidado médico que não necessitasse passar a noite no hospital ou instituição de saúde?

Observation: The underlined terms above refer to the changes that occurred during the stages.

In the pre-test application stage, the interviews were carried out in different states of the Brazilian Northeast, 22 in Rio Grande do Norte (73.3%), four in Ceará (13.3) and four in Paraíba (13.3 %), and had an average duration of 123 minutes. Table 2 contains general information about the characteristics of the pre-test participants.

**Table 2:** Distribution of the pre-test sample according to sociodemographic characteristics (n = 30).

Variables	n or median	% or quartiles (1Q;3Q)
Age (years)^a^	36	28.24; 65.25
Sex		
Female	25	83.3
Male	5	16.7
Marital status		
Single	17	56.7
Married	6	20
Stable union	4	13.3
Separated/divorced	2	6.7
Widower/widow	1	3.3
Schooling		
No education or incomplete primary education	3	10.0
Primary education	2	6.7
Technical education	4	13.3
Secondary education	7	23.3
Higher education	9	30.0
Graduation	5	16.7
Race		
White	8	26.7
Black	4	13.4
Brown	16	53.3
No declaration	1	3.3
Other	1	3.3
Working status		
No employment	7	23.3
Working for an employer and receives a salary	8	26.7
Working and receives a salary, but is currently away for more than three months due to health reasons	2	6.7
Self-employed	2	6.7
Retired due to health conditions	1	3.3
Retired due to age	9	30
Early retirement	1	3.3
Monthly income^a^	R$ 4,750	R$ 2,200; R$ 8,150
Resident per household^a^	3	2; 4

n: sample number; 1Q: first quartile; 3Q: third quartile.

^a^ Values presented in median and quartiles.

Participants mentioned 127 health conditions and most reported having more than one condition, among which the most prevalent were anxiety (14), back pain or herniated disc (13), arthritis or arthrosis (11), hypertension (8), asthma or allergic respiratory disease (8), migraine (8), trouble sleeping (7), gastritis or ulcer (6), tinnitus (6), vision loss (5), depression (5), diabetes (4), and other health conditions (32).

After analyzing the answers from the form related to the participants’ understanding, 63 items needed some adjustment for understanding and, of these, two (I1019, I3033) were sent for analysis by the specialist committee for presenting CVI < 0.80. Such items were submitted to the judge committee and the modifications were made. The items were re-applied to the 30 participants via telephone. The new structure of the items was evaluated by the participants according to a Likert scale of level of understanding, with a CVI > 80 (I1019: CVI 100, I3033: CVI 93.4).

In addition to these, the other items, with errors in grammar, spelling, typing and formatting, were judged by the mediators, observed and adjusted in the questionnaire and in the guide, as recommended by the checklist by Fortes and Araújo^21^ without the need for review by the committee. All adjustments and layouts made in the questionnaire were replicated in the guide and instrument presentation cards, which are available for access on Google drive via link^a^

## DISCUSSION

The use of MDS-Brasil will contribute to surveying the Brazilians’ health needs, knowledge of health conditions, identification of environmental factors, activity limitations, and restrictions on population participation. The comprehensive version of the MDS-Brasil requires application time of around 12 to 15 minutes and should be explored in population surveys, as it is highly capable of generating broad and rich data that aim to improve the lives of persons with different degree disabilities. The summary version (Brief MDS) uses questions selected from the original MDS and can be used as a complementary starting point module for countries interested in developing their own disability modules for household surveys, with an estimated application time of 15 minutes^16^ .

During the process of applying the MDS to the target population, it was observed that the greatest difficulty reported by the participants, and perceived by the research team, was related to the time needed to complete the interview, since the shortest period of time spent was 1 hour and 10 minutes, and the longest 2 hours and 50 minutes. The comments referred to by the sample were based, above all, on the fact that the questionnaire was extensive, tiring and with redundant questions, however it is worth noting that the MDS has a modular format, then the country can choose the modules to be applied, respecting the mandatory modules^13^ , and that the short version is already available for use in surveys^16^ .

Moreover, this application time also includes filling out the ICF and the comprehension questionnaire (total estimated time of 10 minutes). On the other hand, since a tablet application is used during the interview in the population survey and also considering that the interviewer is trained and has experience for its application, it is possible to infer that there will be greater agility to conduct the interview and record the information.

The fact that the sample evaluated in the pre-test was not composed of persons with different disabilities is considered a study limitation, and, therefore, not all questionnaire items were applied.

The psychometric evaluation of the instrument is still necessary, mainly involving populations with disabilities or with specific health conditions^13^ . In this sense, the convergent validity, internal consistencies and other psychometric properties of the Brazilian version of the MDS will be measured in the coming months by the research network.

After analyzing the psychometric properties of the MDS-Brasil, regional and national surveys are expected to be carried out to delineate the profile of functionality and health needs in Brazil from the perspective of the biopsychosocial model, fulfilling one of the applications of the ICF as a social policy tool in the planning of social security systems, compensation systems and the design and implementation of public policies^15^ . Moreover, making the MDS available for use in population data surveys offers an opportunity to generate health indicators on functionality, overcoming the limited approach of mortality and morbidity indicators, contributing to more equitable health care, and allowing the design of collective health interventions that address the real needs of the population^27^ .

## CONCLUSION

The MDS was translated into Brazilian Portuguese and adapted cross-culturally for the Brazilian population with adequate content validity, resulting in the MDS-Brazil version, a WHO tool. The study complied with current international and national operational standards for studies of translation and cross-cultural adaptation of health instruments.

The target audience accepted and understood the Brazilian version of the MDS, which is indicated for population, regional and national surveys, for PWD and people without disabilities.

## Footnotes

a Available at: https://drive.google.com/drive/folders/1B601GPWIb9HksrE1SNMVanZ0TEadJ0qd? .
