# Supplementary material for: Translation and cross-cultural adaptation of the Model Disability Survey (MDS) for Brazil
Source: Rev Saude Publica. 2023 Jun 2;57:33. doi: 10.11606/s1518-8787.2023057004759 (PMC10317131; doi:10.11606/s1518-8787.2023057004759)
Supplement: Translation

# Translation: Tradução e adaptação transcultural do *Model Disability Survey* (MDS) para o Brasil

**Authors:** Érika Giovana Carvalho da Silva, Shamyr Sulyvan Castro, Carla Sabariego, Karolinne Souza Monteiro, Núbia Maria Freire Vieira Lima

**Affiliations:** I Brasil Universidade Federal do Rio Grande do Norte. Faculdade de Ciências da Saúde do Trairi. Santa Cruz, RN, Brasil; II Brasil Universidade Federal do Ceará. Departamento de Fisioterapia. Fortaleza, CE, Brasil; III Switzerland University of Lucerne. Department of Health Sciences and Medicine. Lucerne, Switzerland

**Keywords:** Avaliação da Deficiência, Classificação Internacional de Funcionalidade, Incapacidade e Saúde, Inquéritos e Questionários, Traduções, Comparação Transcultural, Reprodutibilidade dos Testes

## Abstract

**OBJETIVO:**

Traduzir e adaptar transculturalmente o *Model Disability Survey* (MDS), instrumento da Organização Mundial da Saúde que fornece informações abrangentes a respeito de deficiência/funcionalidade, para o Brasil.

**MÉTODOS:**

Trata-se de um estudo metodológico de corte transversal, realizado por meio de cinco etapas – tradução inicial, síntese das traduções, retrotradução, revisão por comitê de especialistas e pré-teste –, considerando propriedades como equivalência semântica, idiomática, experimental e conceitual. Para realização das etapas foram necessários tradutores, pesquisadores, equipe mediadora, profissionais da saúde, metodologista e especialista em idiomas. A análise estatística foi produzida a partir de frequências absolutas e relativas, medidas de tendência central e dispersão, testes de normalidade e índice de validade de conteúdo (IVC) > 0,80.

**RESULTADOS:**

O instrumento MDS apresenta 474 itens, o que gerou 1.896 análises de equivalências. Destes, 160 itens apresentaram IVC < 0,80 em pelo menos uma das quatro equivalências e necessitaram de ajustes. Após adequações e aprovação dos juízes, a versão pré-final seguiu para o pré-teste com 30 participantes, de quatro regiões do Nordeste brasileiro. Desta amostra, 83,3% são mulheres, solteiras, com idade média de 33,7 (DP 18,8) anos, autodeclaradas pretas ou pardas, trabalhadoras ativas, com escolaridade a partir do ensino técnico e que residiam com três moradores. O tempo médio das entrevistas foi de 123 minutos de duração. Foram mencionadas 127 condições de saúde, sendo as mais frequentes ansiedade e dores nas costas. As respostas foram analisadas e 63 itens foram citados como necessitando de algum ajuste, sendo dois destes encaminhados para análise pelo comitê por possuírem IVC < 0,80. O instrumento, manual e cartões de apresentação foram ajustados após um novo pré-teste.

**CONCLUSÕES:**

O MDS foi traduzido e adaptado transculturalmente para o português brasileiro e apresentou adequada validade de conteúdo.

## INTRODUÇÃO

A Organização Mundial da Saúde (OMS), em 2011, estimou que mais de um bilhão de pessoas, o equivalente a 15% da população mundial, têm alguma deficiência^1^ . No Brasil, de acordo com dados da Pesquisa Nacional de Saúde (PNS) de 2019, 17,2 milhões de pessoas com 2 anos ou mais de idade (8,4% da população) possuem algum tipo de deficiência, e destes 8,5 milhões (24,8%) são idosos^2^ .

Mundialmente, os dados existentes sobre deficiência carecem de padronização, pois até o momento não se utiliza um instrumento padrão-ouro para coleta de dados que propicie uma documentação abrangente e sistemática sobre o tema^1 , 3^ . Além disso, compreende-se que o número limitado de perguntas incluídas em censos, como o do Brasil, não é suficiente para fornecer uma medida precisa da quantidade de pessoas com deficiência (PCD), e que, nesse cenário, a utilização de instrumentos padronizados, adaptados transculturalmente e validados é imprescindível para o preenchimento dessa lacuna^4^ .

Nesse sentido, compreende-se que é necessário o desenvolvimento de metodologias padronizadas para a coleta de dados de PCD, em consonância com aspectos culturais e aplicadas de forma consistente, que possibilitem comparações internacionais e permitam o monitoramento de progressos no que se refere às políticas públicas^5^ . O Relatório Mundial sobre a Deficiência ressalta a importância dos países em geral terem conhecimento do número de PCD existentes, bem como seus contextos de vida, visando adequar a prestação de serviços e torná-los mais resolutivos^1^ .

No Brasil e no mundo há instrumentos de medida alinhados ao modelo biopsicossocial, traduzidos e adaptados transculturalmente, que visam mensurar funcionalidade, entre eles o checklist da Classificação Internacional de Funcionalidade, Incapacidade e Saúde (CIF)^6^ , os *Core Sets* , o *World Health Organization Disability Assessment Schedule* (WHODAS 2.0)^7^ e o Índice de Funcionalidade Brasileiro (IFBr).

O checklist da CIF é um instrumento genérico para as condições de saúde que mensura funcionalidade, todavia, a limitação de códigos que podem ser utilizados por cada entrevistado constitui um ponto negativo para sua aplicação^6^ . Os *Core Sets* são listas resumidas dos códigos da CIF com aplicação para condições de saúde específicas, de modo rápido e fácil, contudo, centralizam a avaliação na doença/condição de saúde. O WHODAS 2.0 trata-se de instrumento genérico que avalia a incapacidade percebida associada à condição de saúde, sendo de rápida aplicação. Todavia, não foi criado para inquéritos populacionais e não possui pontos de corte para níveis de deficiência^7^ .

O IFBr é um instrumento proposto pelo governo brasileiro em 2011 com o objetivo de identificar fatores externos que podem influenciar a vida do indivíduo e o quanto podem impactar em sua funcionalidade. O IFBr gera pontuação que quantifica o nível de dependência ou independência funcional do indivíduo em leve, moderada e grave^8^ . Diante da necessidade de algumas modificações no IFBr, foi elaborado o Índice de Funcionalidade Brasileiro Adaptado (IFBrA), o qual é utilizado para avaliar a necessidade da aposentadoria, estando seu uso restrito a pessoas adultas com deficiências ativas no mercado de trabalho^11^ .

Outro ponto a ser destacado é a importância da utilização de uma ferramenta padronizada para levantamento de dados populacionais sobre o impacto da incapacidade na vida das pessoas, evitando assim que ocorra coleta de dados com compreensões destoantes ou equivocadas a respeito da funcionalidade^6^ . Ressalta-se que, principalmente para países com sistemas de saúde baseados na equidade, saber quantas pessoas apresentam deficiências não é suficiente para determinar suas necessidades de saúde; para isso, são necessários dados sobre a incapacidade^12^ .

Diante do exposto, surgiu o *Model Disability Survey* (MDS), a partir de uma parceria entre o Banco Mundial e a OMS^13^ . Tal instrumento apresenta duas versões, abrangente e resumida, ambas de caráter padronizado para coleta de dados em inquéritos em nível populacional, que fornecem informações sobre como as pessoas conduzem suas vidas e as barreiras que encontram, levando em consideração fatores ambientais e pessoais, capacidade e desempenho, permitindo comparações entre grupos com diferentes níveis de funcionalidade^14 , 15^ .

O instrumento MDS já foi implementado por meio de inquéritos nacionais no Chile (2015), Sri Lanka (2015), Filipinas (2017), Catar (2017), Costa Rica (2018) e Afeganistão (2019), regionalmente em Camarões (2016), Paquistão (2017) e Emirados Árabes Unidos (2018), também por meio de estudos piloto em Camboja (2014), Maláiu (2014) e Omã (2016), sendo, portanto, traduzido para os idiomas árabe, espanhol, cingalês, filipino, francês, quemer e dari. No entanto, não foram encontrados estudos que detalhassem os procedimentos de tradução e adaptação transcultural do MDS nesses países^13^ . Em estudo com populações do Chile e do Sri Lanka, o instrumento (versão resumida) revelou métricas válidas para mensurar a deficiência^16^ .

O MDS fundamenta-se na base teórica da CIF^17^ , cuja construção facilita inquéritos de saúde que comparam internacionalmente dados sobre incapacidade ou deficiência^18^ . Os resultados provenientes de inquéritos nacionais permitem e norteiam o planejamento e desenvolvimento de políticas públicas voltadas à plena integração social das PCD^19^ . Nesse contexto, o objetivo desta pesquisa foi realizar a tradução e adaptação transcultural da versão abrangente do MDS para o Brasil, assim como analisar a validade de conteúdo dessa versão brasileira.

## MÉTODOS

Trata-se de um estudo de tradução e adaptação transcultural do instrumento MDS para o português do Brasil, desenvolvido pela Rede de Pesquisa e Inovação em Funcionalidade, Saúde e Objetivos de Desenvolvimento Sustentável (Rede Fusão).

O propósito do MDS é coletar dados sobre todas as dimensões da deficiência de forma a conseguir informações abrangentes e relevantes que ajudem países a construírem um retrato da deficiência, com relevância particular para a política em deficiência; comparações internacionais diretas e confiáveis de dados sobre o assunto; e monitoramento nacional e global da implementação da Convenção Internacional sobre os Direitos das Pessoas com Deficiência. O MDS é subdivido em: questionário sobre o domicílio (folha de rosto, registro de contato, lista de moradores do domicílio), questionário individual (registro do contato, elegibilidade, características sociodemográficas, histórico e benefícios profissionais, fatores ambientais, funcionalidade, condições de saúde, assistência pessoal, dispositivos assistivos e facilitadores, uso de serviços de saúde, bem-estar, empoderamento, observações do entrevistador) e questionário do representante (com os mesmos itens enumerados no módulo anterior)^20^ . O MDS é predominantemente utilizado em populações com 18 anos ou mais, embora também possua um módulo infantil.

O processo de tradução e adaptação transcultural do questionário MDS seguiu as orientações de Fortes e Araújo^21^ e obedeceu às recomendações do *Consensus-based Standards for the Selection of Health Measurement Instruments* (Cosmin)^22^ . Além disso, seguiu as orientações de Beaton e colaboradores^23^ , que fornecem amplo respaldo às exigências semântica, idiomática, experimental e conceitual, estabelecendo cinco etapas, sendo elas: I. Tradução inicial, II. Síntese das traduções, III. Tradução reversa, IV. Revisão por comitê de especialistas e V. Pré-teste.

A primeira tradução (T1 – etapa I) do instrumento MDS foi realizada por empresa especializada em traduções e, em seguida, o documento foi revisado pela equipe da Organização Pan-Americana da Saúde (Opas), representando a tradução realizada com expertise clínica. A segunda tradução (T2) foi feita por dois tradutores leigos, de origem estrangeira e com amplo domínio do português, ambos não informados dos conceitos quantificados pela pesquisa e sem formação na área da saúde.

A síntese das traduções T1 e T2 (versão T12 – etapa II) foi realizada por uma equipe de 10 pesquisadores da área da saúde da Universidade Federal do Rio Grande do Norte (UFRN), sendo eles quatro professoras pesquisadoras, uma discente de mestrado e cinco discentes de iniciação científica. A elaboração da T12 foi realizada via reuniões virtuais, análises e discussões comparativas dos questionários. A fim de garantir a qualidade do processo, todos os itens passaram por revisão por pares.

Na etapa de tradução reversa (etapa III), a versão T12 foi submetida a nova tradução para o idioma inglês (BT1). Esta foi realizada por um tradutor bilíngue, nativo do Canadá, que possui domínio do português, sem conhecimento do instrumento original e dos conceitos explorados na pesquisa.

A etapa IV baseou-se nos critérios de Jasper^24^ para seleção dos especialistas e considerou como composição mínima: metodologistas, profissionais da saúde, profissionais de idiomas e tradutores envolvidos no processo. Nesse sentido, os critérios para seleção foram: ser profissional com conhecimento sobre funcionalidade, deficiência ou sobre o processo de tradução e validação de instrumentos de medida em saúde. Os especialistas foram selecionados de diferentes estados brasileiros por meio de consulta ao Currículo Lattes do Conselho Nacional de Desenvolvimento Científico e Tecnológico (CNPq), formando o comitê com quatro juízes (sendo um deles metodologista e todos profissionais da área da saúde) e uma especialista em idiomas. Os juízes receberam sete documentos com as versões T1, T2, T12, BT1, instrumento MDS em inglês, manual do MDS em inglês e português, cronograma das reuniões e demais orientações relacionadas às equivalências. Esses profissionais realizaram a leitura e análise completa dos documentos com auxílio da comissão mediadora (duas professoras e uma discente de mestrado da UFRN, que participaram das etapas anteriores) e as divergências residuais foram enviadas para a especialista em idiomas. Essa equipe de mediação tinha como papel principal nortear o comitê de juízes ao decorrer da etapa, planejar e executar reuniões virtuais estratégicas, assim como realizar todos os ajustes e adequações necessários nos documentos de acordo com as orientações do comitê.

Cada item do questionário foi avaliado por meio de uma ferramenta elaborada nas Planilhas Google, que viabilizou as análises, compartilhadas individualmente com cada juiz. Essa ferramenta incluiu todos os itens do instrumento original e da versão T12, subdivido de acordo com os módulos do MDS, com campos de caráter qualitativo e quantitativo, sendo, respectivamente, um para considerações sobre as traduções, adequação dos itens e possíveis sugestões; e outro para classificar as equivalências semântica, idiomática, experimental e conceitual como tradução adequada (TA), tradução parcialmente adequada (TPA) e tradução inadequada (TI).

No processo de avaliação individual dos itens, foi considerado o índice de validade de conteúdo (IVC) maior que 80%^25^ , que se refere ao grau em que o conteúdo de um instrumento reflete adequadamente o construto que está sendo medido. O IVC é baseado na avaliação de especialistas para cada item de acordo com a relevância do conteúdo de um instrumento, julgado geralmente por meio de escalas Likert^26^ . Como o comitê contou com a participação de quatro membros (25% por membro)^25^ , quando um dos juízes discordou a respeito de um item, este foi considerado alvo de alguma revisão.

As sugestões realizadas pelos juízes foram agrupadas, junto das respectivas justificativas, e ajustadas de acordo com o consenso dos juízes. Por fim, foi realizado um seminário integrativo, com os membros dessa etapa, por meio de reunião virtual para análise do instrumento em sua versão pré-final e ajustes antes do pré-teste.

Para a etapa de pré-teste (etapa V), os critérios de inclusão de participantes foram: pessoas maiores de 18 anos e com capacidade cognitiva de responder ao questionário. Os critérios de exclusão adotados foram: negar-se a responder a todas as perguntas do questionário e desistir da entrevista antes de ser finalizada.

A amostra foi composta por 30 participantes, selecionada por conveniência de acordo com os critérios de elegibilidade e a localidade dos entrevistadores. Os participantes foram convidados por contato telefônico e a entrevista foi realizada de forma presencial, em sala reservada. A coleta foi feita por oito membros da equipe do projeto de pesquisa, composto por docentes e discentes dos mestrados da UFRN, que receberam treinamento prévio para realização das entrevistas e possuíam conhecimento sobre a temática do estudo.

As respostas ao questionário foram enviadas pelos entrevistadores para um banco de dados on-line, por meio de Formulários Google previamente elaborados para esta etapa, além de um específico para avaliar a compreensão do entrevistado. Esse formulário foi utilizado para registro dos itens que, por autorrelato do participante ou por percepção de dificuldade observada durante a entrevista, necessitavam de ajustes para melhorar o entendimento. Todos os entrevistadores foram orientados a aplicar as perguntas do formulário de compreensão ao final de cada módulo do MDS.

Após a finalização da coleta da etapa V, todos os itens citados no formulário de compreensão foram analisados. Para essa análise também foi considerado o IVC: % concordância=número de participantes que concordaram/número total de participantes*100. Ou seja, 80%=X/30*100.

Nesse sentido, quando houve sete ou mais citações de discordância a respeito de um item no formulário de compreensão (IVC < 80), este passou por nova análise do comitê de especialistas. Os itens ajustados foram avaliados pelos 30 participantes por meio de nova etapa de pré-teste, realizada apenas com esses itens pendentes, de acordo com uma escala Likert de nível de compreensão, com as seguintes alternativas de respostas: 1. Muito boa, 2. Boa, 3. Regular, 4. Ruim, 5. Muito ruim. Nessa etapa, caso houvesse sete ou mais citações por item elencados como “Ruim” ou “Muito ruim”, este era reanalisado. Como a nova realização do pré-teste se deu apenas com os itens faltantes, foi permitido aos entrevistadores a realização dessa etapa via contato telefônico, visando facilitar o processo de coleta.

O resumo de todas as etapas de tradução e adaptação transcultural do MDS está contido na Figura .

**Figura:** Fluxograma do processo de tradução e adaptação transcultural do *Model Disability Survey* para o português brasileiro (MDS-Brasil).

O manual do MDS^20^ e o arquivo dos cartões de apresentação também passaram por processo de tradução, feito por profissionais naturais do Brasil com ampla experiência em traduções e professores de língua portuguesa, e posteriormente foram encaminhados para o comitê de especialistas. Portanto, passaram por um processo semelhante ao do instrumento abrangente.

Todos os dados coletados foram inseridos em um banco de dados, elaborado por meio do Microsoft Excel^®^, versão 2016, e analisados com o programa *Statistical Package for the Social Science* (SPSS for Windows *)* ^®^, versão 25.0. A distribuição da amostra foi apresentada por meio de frequência absoluta e relativa, e a análise descritiva foi realizada utilizando as medidas de tendência central e dispersão, sendo mediana e quartis respectivamente. Para verificar a normalidade de distribuição das variáveis quantitativas, foi aplicado o teste de normalidade de Shapiro-Wilk. O IVC foi utilizado durante as etapas IV e V para medir a proporção ou porcentagem de concordância sobre os itens do instrumento e estabelecer que as etapas fossem realizadas até todos os itens, ou conjunto de itens, atingirem concordância > 0,80^26^ .

Todos os participantes da etapa V foram informados sobre a pesquisa e autorizaram a participação através da assinatura do Termo de Consentimento Livre e Esclarecido (TCLE). O estudo cumpriu os preceitos éticos que regem a pesquisa com seres humanos conforme Resolução nº 466/12 do Conselho Nacional de Saúde (CNS), e especificado na Declaração de Helsinque, com a aprovação do Comitê de Ética em Pesquisa Institucional da Faculdade de Ciências da Saúde do Trairi (Facisa) sob o n. 4.102.958 e Certificado de Apresentação de Apreciação Ética (Caae) n. 31112020.4.0000.5568.

## RESULTADOS

Durante a etapa do comitê de especialistas, o instrumento MDS foi considerado com totalidade de 474 itens, na qual também foi incluída a contagem dos enunciados e títulos dos módulos. No entanto, como cada item foi analisado de acordo com quatro equivalências (semântica, idiomática, experimental e conceitual), houve um total de 1.896 análises por juiz. A Tabela 1 faz referência a essas análises de concordâncias individuais realizadas pelos juízes durante a etapa IV.

**Tabela 1:** Análise quantitativa de concordância dos itens verificados pelos especialistas.

Especialista	TA		TPA		TI	
	n	%	n	%	n	%
Juiz 1	1.857	97,94%	37	1,95%	2	0,11%
Juiz 2	1.773	93,51%	99	5,22%	24	1,27%
Juiz 3	1.827	96,36%	66	3,48%	3	0,16%
Juiz 4	1.800	94,94%	81	4,27%	15	0,79%

TA: tradução adequada; TPA: tradução parcialmente adequada; TI: tradução inadequada.

Um total de 327 equivalências (17,25%) foram julgadas como parcialmente adequadas e inadequadas pelos especialistas. Considerando apenas os itens divergentes, ou seja, aqueles nos quais pelo menos uma das quatro equivalências foi classificada como TPA ou TI por pelo menos um juiz, obteve-se um total de 160 itens encaminhados para discussão com os demais membros do comitê de juízes para deliberação e consenso da versão pré-final.

Em razão do grande número de itens presentes no questionário MDS, o Quadro ilustra a descrição de parte daqueles que passaram por alguma alteração durante o estágio final da etapa IV (n = 160), em comparação com a versão original e a versão síntese das traduções (T12). As modificações realizadas estão sublinhadas para melhor visualização.

**Quadro:** Comparação das mudanças nos itens entre a versão original e versão adaptada do *Model Disability Survey* (MDS) para uso no Brasil.

Item	Versão original	Síntese das traduções (T12)	Versão pré-final aprovada pelo comitê de juízes para aplicação no pré-teste
Módulo 0200, alternativa D	Contact with	Em contato com:	Fez contato com:
Módulo 1000, do Questionário Domiciliar	Household roster	Descrição do domicílio	Lista de moradores do domicílio
Módulo 2000, Questão 2013, do Questionário Individual	Do/did you usually work throughout the year, or do/did you work seasonally, or only once in a while for your main job? Work throughout the year Seasonally or part of the year Once in a while	Em relação ao seu trabalho principal, você costuma/costumava trabalhar durante todo o ano ou apenas em determinadas temporadas ou durante pequenos períodos? Durante todo o ano Determinadas temporadas Ocasionalmente (durante pequenos períodos)	Em relação ao seu trabalho principal, você costuma/costumava trabalhar durante todo o ano, por determinadas temporadas ou durante pequenos períodos? Durante todo o ano Por temporada Ocasionalmente (durante pequenos períodos)
Módulo 4000, Questão 4010, do Questionário Individual	How much of a problem is being clean and dressed?	O quanto manter sua higiene pessoal e vestir-se é um problema para você?	O quanto fazer sua higiene pessoal e vestir-se é um problema para você?
Segundo enunciado do Módulo 4000, do Questionário Individual	Please take into account your health and people who help you, any assistive devices you use or any medication you take.	Por favor, considere a sua saúde e as pessoas que te ajudam, quaisquer dispositivos de auxílio que utiliza ou quaisquer medicamentos que você toma.	Por favor, considere a sua saúde e as pessoas que te ajudam, quaisquer dispositivos assistivos que utiliza ou quaisquer medicamentos que você toma.
Módulo 3000B, do Questionário Individual	Mobility and Self-care	Mobilidade e Cuidado Pessoal	Mobilidade e Autocuidado
Módulo l6010, do Questionário Individual	Over the last 12 months, did you receive any health care NOT including an overnight stay in hospital, rehabilitation facility or long-term care facility?	Nos últimos 12 meses, você recebeu algum cuidado médico que NÃO necessitaram passar a noite no hospital ou instituição de saúde?	Nos últimos 12 meses, você recebeu algum cuidado médico que não necessitasse passar a noite no hospital ou instituição de saúde?

Nota: Os termos sublinhados acima referem-se às mudanças ocorridas no decorrer das etapas.

Na etapa de aplicação do pré-teste, as entrevistas foram realizadas em diferentes estados do Nordeste brasileiro, sendo 22 no Rio Grande do Norte (73,3%), quatro no Ceará (13,3) e quatro na Paraíba (13,3%), e tiveram um tempo médio de 123 minutos de duração. Na Tabela 2 constam as informações gerais acerca das características dos participantes do pré-teste.

**Tabela 2:** Distribuição da amostra do pré-teste de acordo com características sociodemográficas (n = 30).

Variáveis	n ou mediana	% ou quartis (1Q;3Q)
Idade (anos)^a^	36	28,24; 65,25
Sexo		
Feminino	25	83,3
Masculino	5	16,7
Estado civil		
Solteiro	17	56,7
Casado	6	20
União estável	4	13,3
Separado/divorciado	2	6,7
Viúvo	1	3,3
Escolaridade		
Sem escolaridade ou Ensino Fundamental Incompleto	3	10,0
Ensino Fundamental	2	6,7
Ensino Técnico	4	13,3
Ensino Médio	7	23,3
Ensino Superior	9	30,0
Pós-Graduação	5	16,7
Raça		
Branca	8	26,7
Preta	4	13,4
Parda	16	53,3
Não quis declarar	1	3,3
Outro	1	3,3
Situação atual de trabalho		
Desempregado	7	23,3
Trabalha para um empregador e recebe pagamento em salário	8	26,7
Trabalha e recebe pagamento, mas atualmente está afastado há mais de três meses por motivos de saúde	2	6,7
Profissional autônomo	2	6,7
Aposentado por condições de saúde	1	3,3
Aposentado devido à idade	9	30
Aposentadoria precoce	1	3,3
Renda mensal^a^	R$ 4.750	R$ 2.200; R$ 8.150
Morador por domicílio^a^	3	2; 4

n: número amostral; 1Q: primeiro quartil; 3Q: terceiro quartil.

^a^Valores apresentados em mediana e quartis.

Foram citadas 127 condições de saúde pelos participantes e a maioria informou possuir mais de uma condição, dentre elas as mais prevalentes foram ansiedade (14), dores nas costas ou hérnia de disco (13), artrite ou artrose (11), hipertensão (8), asma ou doença respiratória alérgica (8), enxaqueca (8), problemas para dormir (7), gastrite ou úlcera (6), zumbido (6), perda da visão (5), depressão (5), diabetes (4) e outras condições de saúde (32).

Após a análise das respostas do formulário de compreensão dos participantes, 63 itens necessitaram de algum ajuste para o entendimento e, destes, dois (I1019, I3033) foram encaminhados para análise do comitê de especialistas por possuírem IVC < 0,80. Tais itens foram encaminhados ao comitê de juízes e as modificações foram executadas. Os itens foram reaplicados nos 30 participantes via contato telefônico. A nova estrutura dos itens foi avaliada pelos participantes de acordo com uma escala Likert de nível de compreensão, sendo obtido IVC > 80 (I1019: IVC 100, I3033: IVC 93,4).

Além destes, os demais itens, com erros de gramática, ortografia, digitação e formatação, foram julgados pelos mediadores, observados e ajustados no questionário e no manual, conforme preconizado pelo checklist de Fortes e Araújo^21^ sem necessidade de revisão pelo comitê. Todos os ajustes e diagramações realizados no questionário foram replicados no manual e cartões de apresentação do instrumento, que estão disponíveis para acesso através do link do drive^a^ .

## DISCUSSÃO

O uso do MDS-Brasil contribuirá para o levantamento das necessidades em saúde dos brasileiros, o conhecimento de condições de saúde, a identificação de fatores ambientais, a limitação de atividades e as restrições de participação populacional. A versão abrangente do MDS-Brasil demanda tempo de aplicação em torno de 120 a 15 minutos e deve ser explorada em inquéritos populacionais, pois é altamente capaz de gerar dados amplos e ricos que visam melhorar a vida de pessoas com deficiências em diferentes graus. Já a versão resumida ( *Brief* MDS) utiliza perguntas selecionadas do MDS original e pode ser utilizado como um módulo complementar de ponto de partida para países interessados em desenvolver seus próprios módulos de deficiência para pesquisas domiciliares, com um tempo estimado de aplicação de 15 minutos^16^ .

Durante o processo de aplicação do MDS na população-alvo, foi observado que a maior dificuldade relatada pelos participantes, e percebida pela equipe de pesquisadores, estava relacionada ao tempo necessário para conclusão da entrevista, visto que o menor tempo gasto foi de 1 hora e 10 minutos e o maior 2 horas e 50 minutos. Os comentários referidos pela amostra se pautavam, sobretudo, no fato do questionário ser extenso, cansativo e com perguntas redundantes, entretanto é válido ressaltar que o MDS tem formato modular, portanto o país pode escolher os módulos a serem aplicados, respeitando os módulos obrigatórios^13^ , e que a versão breve também já está disponível para uso em inquéritos^16^ .

Além disso, esse tempo de aplicação também contempla o uso associado do TCLE e do questionário de compreensão (tempo estimado total de 10 minutos). Por outro lado, uma vez que durante a entrevista no inquérito populacional seja utilizado um aplicativo de tablet e considerando também que o entrevistador tenha treinamento e experiência para sua aplicação, acredita-se que haverá maior agilidade para conduzir a entrevista e registrar as informações.

Uma limitação deste estudo se pauta no fato de que a amostra avaliada no pré-teste não foi composta por pessoas com variadas deficiências, portanto, nem todos os itens do questionário foram aplicados.

Ainda é necessária a avaliação psicométrica do instrumento, principalmente envolvendo populações com deficiências ou com condições de saúde específicas^13^ . Nesse sentido, a validade convergente, as consistências internas e as demais propriedades psicométricas da versão brasileira do MDS serão mensuradas nos próximos meses pela rede de pesquisa.

Após a análise das propriedades psicométricas do MDS-Brasil, aguarda-se a execução de inquéritos regionais e nacionais para delineamento do perfil de funcionalidade e necessidades em saúde no Brasil sob a perspectiva do modelo biopsicossocial, cumprindo uma das aplicações da CIF como ferramenta de política social no planejamento dos sistemas de previdência social, sistemas de compensação e projeto e implementação de políticas públicas^15^ . Além disso, a disponibilização do MDS para uso no levantamento de dados populacionais oferece oportunidade de geração de indicadores de saúde sobre a funcionalidade, superando a abordagem limitada de indicadores de mortalidade e morbidade, contribuindo para um cuidado em saúde mais equitativo e permitindo o desenho de intervenções coletivas em saúde que contemplem as reais necessidade da população^27^ .

## CONCLUSÃO

Foi realizada a tradução do MDS para o português do Brasil, adaptando o instrumento transculturamente para a população brasileira com adequada validade de conteúdo, resultando na versão MDS-Brasil, ferramenta da OMS. O estudo cumpriu as normas operacionais internacionais e nacionais vigentes para estudos de tradução e adaptação transcultural de instrumentos em saúde.

Verificou-se aceitação e compreensão do público-alvo da versão brasileira do MDS, que é indicado para inquéritos populacionais, regionais e nacionais, para PCD e pessoas sem deficiências.

## Footnotes

a Disponível em: https://drive.google.com/drive/folders/1B601GPWIb9HksrE1SNMVanZ0TEadJ0qd? .
